# The accuracy of chemotherapy ascertainment among colorectal cancer patients in the surveillance, epidemiology, and end results registry program

**DOI:** 10.1186/s12885-018-4405-7

**Published:** 2018-04-27

**Authors:** Mark A. Healy, Arden M. Morris, Paul Abrahamse, Kevin C. Ward, Ikuko Kato, Christine M. Veenstra

**Affiliations:** 1 Center for Healthcare Outcomes & Policy, University of Michigan, 300 North Ingalls, Rm 3A22, Ann Arbor, MI 48105 USA; 2 Institute for Healthcare Policy and Innovation, University of Michigan, 300 North Ingalls, Rm 3A22, Ann Arbor, MI 48105 USA; 3 Department of Epidemiology, Rollins School of Public Health, Emory University, Atlanta, GA USA; 4 Department of Pathology, Wayne State University, Detroit, MI USA

**Keywords:** SEER, Colorectal cancer, Chemotherapy, Registries

## Abstract

**Background:**

Surveillance, Epidemiology, and End Results (SEER) public research database does not include chemotherapy data due to concerns for incomplete ascertainment. To compensate for perceived lack of data quality many researchers use SEER-Medicare linked data, limiting studies to persons over age 65. We sought to determine current SEER ascertainment of chemotherapy receipt in two relatively large SEER registries compared to patient-reported receipt and to assess patterns of under-ascertainment.

**Methods:**

In 2011–14, we surveyed patients with Stage III colorectal cancer reported to the Georgia and Metropolitan Detroit SEER registries. 1301/1909 eligible patients responded (68% response rate). Survey responses regarding treatment and sociodemographic factors were merged with SEER data. We compared patient-reported chemotherapy receipt with SEER recorded chemotherapy receipt. We estimated multivariable regression models to assess associations of under-ascertainment in SEER.

**Results:**

Eighty-five percent of patients reported chemotherapy receipt. Among those, 10% (*n* = 104) were under-ascertained in SEER (coded as not receiving chemotherapy). In unadjusted analyses, under-ascertainment was more common for older patients (11.8% age 76+ vs. < 9% for all other ages, *p* = 0.01) and varied with SEER registries (10.2% Detroit vs. 6.8% Georgia; *p* = 0.04). On multivariable analyses, chemotherapy under-ascertainment did not vary significantly by any patient attributes.

**Conclusion:**

We found a 10% rate of under-ascertainment of adjuvant chemotherapy for resected, stage III colorectal cancer in two SEER registries. Chemotherapy under-ascertainment did not disproportionately affect any patient subgroups. Use of SEER data from select registries is an important resource for researchers investigating contemporary chemotherapy receipt and outcomes.

## Background

Since its inception in 1973, the National Cancer Institute’s Surveillance, Epidemiology, and End Results (SEER) program has collected data of critical importance to cancer epidemiology, policy, and health services research. Today, SEER data cover 30% of the US population, [1] and have informed over 7000 published studies. The population-based nature of these data afford the opportunity to examine cancer care delivery and explore outcomes in patients beyond those treated at individual cancer centers or groups of centers. Chemotherapy ascertainment in the SEER database has been considered unreliable, [2, 3] however, limiting its use as a stand-alone database for one of the most important aspects of cancer treatment.

Consequently, most population-based studies of chemotherapy receipt have relied on claims linkages, specifically the SEER-Medicare linked dataset, and therefore have been restricted to patients aged 65 years and older. [4–10] While a cancer diagnosis is more common among the elderly, SEER-Medicare-based studies overlook those under age 65, resulting in very limited knowledge about cancer treatment among the 83% of U.S. adults who are aged 19–64 years. As well, studies using SEER-Medicare data are limited by an extended delay in release relative to SEER data alone because the SEER-Medicare datasets are only created every other year.

The potential for under-ascertainment of chemotherapy by SEER registries is real, especially with the increasing use of oral oncolytics and other chemotherapies administered outside of the hospital setting that can be difficult for registrars to capture. If under-ascertainment is prevalent or varies by patient sociodemographic, clinical, or geographic factors, then studies that use SEER data alone to investigate chemotherapy risk inaccurate conclusions that could spur unnecessary changes in clinical practice or health policy. If under-ascertainment of chemotherapy is not a significant problem, however, SEER data could inform studies that would not be limited to elderly patients only.

To further explore the current utility of SEER data as a stand-alone source for population-based studies of chemotherapy, we compared patient-reported to SEER Registry-reported receipt of adjuvant chemotherapy from two diverse population-based registries. We specifically surveyed patients with Stage III colorectal cancer, the third most common cancer diagnosis among men and women in the United States. [11] Among patients with node positive, locally advanced disease, six months of fluoropyrimidine-based adjuvant chemotherapy is the standard recommendation following surgical resection [12–14] as reflected in the National Comprehensive Cancer Network (NCCN) clinical guidelines. [15] Despite this recommendation, multiple population-based studies have shown that more than 30% of patients do not receive adjuvant chemotherapy, with notable racial and socioeconomic disparities in chemotherapy receipt. [16–19] In our study we sought to answer the following questions: 1) How well do select SEER registries ascertain adjuvant chemotherapy receipt in the current era? and 2) Are there specific demographic subpopulations for whom chemotherapy receipt is systematically under-ascertained?

## Methods

### Study overview

As previously detailed, [20] we identified all patients aged ≥18 years who underwent surgical resection for pathologic stage III colon or rectal cancer between August 1, 2011 and December 31, 2013 and were reported to the Surveillance, Epidemiology, and End Results (SEER) cancer registries at two representative sites, Metropolitan Detroit, Michigan and the State of Georgia. Patients were identified through rapid case ascertainment at the registries utilizing virtual real-time pathology reports and were eligible for recruitment starting 4 months after diagnosis, when chemotherapy should have initiated. [21–24]

### Data collection

Our data collection procedures have been detailed previously. [25] Briefly, we used a modified version of the Dillman approach for recruitment, including a small incentive payment. [26] A research information sheet in the survey packet included language regarding the study purpose, risks and benefits of participation, and patient confidentiality. The return of a completed survey was considered implied consent to participate in the study. Survey responses were accepted up to 1 year from the date of surgery; the last day to accept survey responses was December 31, 2014. The SEER Registries supplemented patient-reported data with clinical information from the registry and census tract-level area-based measures of socioeconomic status (SES).

The study protocol was approved by the institutional review boards of the University of Michigan, Wayne State University, Emory University, the State of Michigan, and the State of Georgia Department of Public Health.

### Measures

We defined the primary dependent variable, under-ascertainment of chemotherapy, as a Boolean variable where true was defined by positive patient-reported receipt of chemotherapy and SEER-coded non-receipt of chemotherapy. We defined a secondary outcome, over-ascertainment of chemotherapy, as a Boolean variable where true was defined as negative patient-reported receipt of chemotherapy and SEER-coded receipt of chemotherapy. We measured chemotherapy receipt by asking: “Did you or are you going to have chemotherapy to treat your colorectal cancer?” The accuracy of self-report of cancer treatments, including chemotherapy, has been validated in previous studies. [27–29] We also asked about the timing of treatment. Those who reported that they planned to receive chemotherapy but had yet to start were excluded from analysis (*n* = 34), so that the self-reported measure of chemotherapy receipt in this study was considered positive only for patients who reported already receiving chemotherapy treatment.

Independent variables included age, gender, marital status, race, comorbid conditions, insurance status, annual household income, educational attainment, and area-level SES index (principle component analysis of area-level high school degree, college degree, and poverty level combined into a composite standardized measure of the economic environment). Among the 19% of cases missing patient-reported annual household income, we performed multiple imputation. Values for missing income were imputed using sequential multiple imputation. Five multiply imputed datasets were analyzed and the results combined to account for additional uncertainty due to imputation. [30] These results were compared with model results from the non-imputed dataset for any meaningful differences.

We assessed primary disease site (colon versus rectum) via the SEER registry using ICD-O-3 Site codes (colon: C180–189; rectosigmoid junction/rectum: C199, C209) and excluding ICD-O-3 histology codes 9050–5, 9140, and 9590–9992. SEER registries used data from the American Hospital Association Database to identify the hospital where the colorectal cancer surgery was performed, and we categorized hospitals based on bed size collapsed into tertiles. We also ascertained the hospital nurse:bed ratio (< 1.0, 1.0–1.49, 1.5–1.99, ≥ 2.0), Joint Commission on Accreditation of Healthcare Organizations accreditation, American College of Surgeons cancer program, Accreditation Council for Graduate Medical Education residency training program, medical school affiliation, and Council of Teaching Hospitals status (all yes/no) but did not include these variables in our final analyses as they were not significantly associated with under-ascertainment in either bivariate or multivariable analyses.

To determine the chemotherapy receipt status in registry data, we used the “RX_SUMM_CHEMO” variable, which indicates any receipt of chemotherapy as part of initial therapy in SEER (reference SEER Program Code Manual). SEER defines adjuvant chemotherapy as postoperative first-course chemotherapy which ends when the documented treatment plan is completed or at disease progression, recurrence, or treatment failure. Individuals coded as 00 (none) or 85–87 (chemotherapy was not administered) were categorized as not receiving chemotherapy; those assigned codes 01–03 (codes for chemotherapy with single agent, multiagent and unknown number of agents) were categorized as receiving chemotherapy. The 4% who were coded as 88 (planned, unknown if given) and 99 (unknown if administered) were excluded from analyses of under-ascertainment.

### Analysis

We compared patient-reported and SEER registry-reported receipt of chemotherapy. We then described the frequency of chemotherapy under-ascertainment at each SEER site after grouping patients by clinical and sociodemographic characteristics, as well as by treatment and hospital characteristics.

Univariate analyses were performed using Pearson’s χ^2^ tests. We then regressed registry under-ascertainment on age, sex, race, marital status, number of comorbid conditions, insurance status, primary tumor site, and SEER region, adjusting for clustering by hospital. Because none of the hospital attributes were significantly associated with under-ascertainment, we omitted these from the final regression models. We evaluated all first-order interactions between significant variables; none were significant except as reported. Survey nonresponse was significantly greater among older, nonwhite, and rectal cancer patients. To adjust for this, response weights were created as inverse probability weights derived from a logistic regression of survey response. The weights were normalized to equal the observed sample size and were incorporated in all multivariable models. [31] Results are presented as unweighted values, with weighted percentages. All analyses were performed with SAS 9.4 software (SAS Inc., Cary, North Carolina).

## Results

### Study sample and response rate

We identified 2168 patients with Stage III colorectal cancer reported to the SEER registries of Georgia and Detroit, Michigan using rapid case ascertainment. Among these, 259 (12%) were later determined to be ineligible (non-Stage III disease, non-colorectal primary, prior cancer diagnosis, or residing outside the registry area). Of the 1909 eligible patients included in the final sample, 608 could not be located or did not return the survey, leaving a sample of 1301 patients (68% survey response rate). Of these patients, 48 had missing data in SEER regarding chemotherapy receipt. Thus, our analytic sample was comprised of 1253 patients.

Among the entire analytic sample, 1068 patients self-reported receipt of adjuvant chemotherapy (85% chemotherapy receipt rate). Among patients who self-reported receipt of chemotherapy, 104 (10%) were coded as not receiving chemotherapy in SEER (under-ascertained). Among patients who self-reported non-receipt of chemotherapy, 33 (18%) were coded as receiving chemotherapy in SEER. (Table 1). The sensitivity of SEER to identify patients who received chemotherapy was 90%; specificity was 82%. The positive predictive value of SEER was 97%, and the negative predictive value was 59%.

**Table 1:** Patient self-report of chemotherapy receipt compared With SEER registry data

SEER status	Patient reports chemotherapy	Patient reports no chemotherapy
SEER records chemotherapy	964	33
SEER records no chemotherapy	104	152
Total	1068	185

*SEER* Surveillance, Epidemiology, and End Results

In univariate analyses, under-ascertainment was significantly associated with older age (12% age 76+ vs. < 9% for all other ages, *P* = 0.01) and in patients living in Detroit compared with the State of Georgia (11% vs. 7%; *P* < 0.04) (Table 2). In multivariable analyses, chemotherapy under-ascertainment was not associated with any patient attributes (Table 3). Variable selection techniques were used to examine models using subsets of the covariates but none of these affected the significance of the covariates or substantially improved the overall fit of the model. No meaningful differences were noted in non-imputed analyses.

**Table 2:** Patient characteristics and unadjusted under-scertainment of chemotherapy by the SEER registry

Characteristic	No.	Weighted %	% Under-ascertained^a^	*P* ^*b*^
Age, years				0.01
< 50	219	16.8	3.6	
50–64	469	36.1	7.5	
65–74	302	23.2	8.4	
75+	311	23.9	11.8	
Sex				0.45
Female	601	46.7	8.9	
Male	685	53.3	7.7	
Marital Status				0.61
Single	545	41.9	8.6	
Married	756	58.1	7.8	
Comorbid Conditions				0.42
None	319	24.5	6.7	
1	398	30.6	9.5	
2+	584	44.9	8.0	
Race				0.24
White	889	69.0	7.4	
Black	327	25.4	10.5	
Other	73	5.7	8.9	
Census Tract Composite SES				0.20
High	352	27.1	7.7	
Medium	498	38.4	6.9	
Low	448	34.5	10.0	
Insurance				0.12
None	112	8.8	3.5	
Medicaid	51	4.0	11.4	
Medicare	578	45.3	9.9	
Other	536	42.0	7.0	
Primary Disease Site				0.06
Colon^c^	985	75.7	9.3	
Rectum	316	24.3	5.6	
Geographic Site				0.04
Detroit	475	36.5	10.2	
Georgia	826	63.5	6.8	
Hospital Bed Size				0.70
< 300	450	33.8	8.2	
300–499	318	24.7	7.0	
≥500	533	41.5	8.7	
ACS Cancer Program				0.90
Yes	957	75.3	8.2	
No	314	24.7	8.0	

^a^Percentage under-ascertained calculated within the weighted sample. ^b^*P* values for differences in the proportion of chemotherapy under-ascertainment by the categories shown. ^c^Rectosigmoid primary tumor sites are included within “Colon”

*SES* socioeconomic status

*ACS* American College of Surgeons

**Table 3:** Multivariable logistic regression models of chemotherapy under-ascertainment

Characteristic	Odds Ratio	95% Confidence Interval	*P*
Age, years			0.08
< 50	Ref	Ref	
50–64	2.14	0.96–4.79	
65–74	2.12	0.84–5.37	
75+	3.12	1.27–7.70	
Race			0.28
White	Ref	Ref	
Black	1.51	0.91–2.51	
Hispanic	1.38	0.16–11.56	
Socioeconomic Status	1.01	0.78–1.31	0.93
Insurance			0.54
Other	Ref	Ref	
None	0.47	0.13–1.67	
Medicaid	1.50	0.52–4.35	
Medicare	1.07	0.59–1.96	
Geographic Site			0.14
Detroit	Ref	Ref	
Georgia	0.71	0.46–1.12	
Hospital Bed Size			0.75
< 100	Ref	Ref	
300–499	0.86	0.49–1.51	
≥500	1.06	0.64–1.76	

We present here the covariates of most interest; the multivariable regression model was adjusted for all covariates (age, sex, marital status, comorbid conditions, race, composite socioeconomic status, insurance, primary disease site, geographic site, hospital bed size, and American College of Surgeons cancer program status). Covariates not shown in the table were not significantly associated with under-ascertainment of chemotherapy receipt

## Discussion

We have shown a 10% rate of under-ascertainment of adjuvant chemotherapy among patients with resected, stage III colorectal cancer in the SEER registries participating in this study. In multivariable analyses, under-ascertainment did not vary by patient sociodemographic factors.

A recent study comparing rates of chemotherapy ascertainment in SEER to those in SEER-Medicare reported a relatively low sensitivity of chemotherapy ascertainment in SEER, and cautioned against the use of SEER for studies of chemotherapy use. [2] However, our study differs from that study in important ways. Due to the use of SEER-Medicare data as the criterion for ascertaining receipt of chemotherapy, the authors did not include patients under age 65. Furthermore, the study population included only patients diagnosed between 2000 and 2006, and therefore does not reflect more recent trends in chemotherapy receipt. That study, focused on all treatments (radiation, chemotherapy, and hormone therapy) across all stages of multiple cancers, did not restrict their analyses to patients who, according to published guidelines, should have received selected therapies. Thus, the authors reported very low rates of chemotherapy receipt across several cancers, including colorectal cancer, and reported low sensitivity of SEER to ascertain chemotherapy receipt for colorectal cancer when compared with SEER-Medicare (71.4% sensitivity, 95% CI 70.8–72.0).

In contrast, 85% of our study respondents reported receipt of adjuvant chemotherapy for Stage III colorectal cancer, a cancer in which clear guidelines for the use of adjuvant chemotherapy exist. We found high sensitivity of SEER to identify patients who received chemotherapy (90% sensitivity) and a high positive predictive value (97%), indicating that among those identified in SEER as receiving chemotherapy, the vast majority also self-reported receipt of chemotherapy. Our study includes younger patients and is therefore more representative of the total population of colorectal cancer patients. Additionally, we were able to include patients with both Medicare and non-Medicare health insurance and found no significant differences in chemotherapy ascertainment by insurance type. We included patients diagnosed between 2011 and 2013, providing a view into modern use of adjuvant chemotherapy. In recent years adjuvant chemotherapy for stage III colon cancer has been one of the most widely accepted and utilized quality of care measures [22, 32] and our findings suggest that receipt of adjuvant chemotherapy has improved significantly over the past decade. Furthermore, we relied upon patient-report of chemotherapy receipt as the criterion to which we compared SEER ascertainment of chemotherapy, rather than alternate registry data that could also risk under-ascertainment. Therefore, we believe that we present a current picture of adjuvant chemotherapy receipt as reported by patients across the age spectrum. The relatively low under-ascertainment of 10%, in conjunction with the finding that chemotherapy under-ascertainment did not disproportionately affect patient subgroups, suggests that SEER data may be useful in focused studies of chemotherapy for select cancers and/or registries.

Because the concern for under-ascertainment of chemotherapy in SEER has limited its use as a stand-alone database for population-based studies of chemotherapy, we focused our study on under-ascertainment as the primary outcome. To expand upon the understanding of the accuracy of chemotherapy ascertainment in SEER, however, we looked at possible causes of chemotherapy over-ascertainment in our study population as a secondary outcome. We found that most patients who self-reported that they did not receive chemotherapy were prescribed oral capecitabine. A few patients started chemotherapy but stopped after one dose, and it is possible that some patients had not yet started chemotherapy at the time they completed the survey, answered “no” to the survey item regarding receipt of chemotherapy, but then started chemotherapy shortly afterwards.

There are several ways in which SEER registries can improve the accuracy of chemotherapy ascertainment. SEER registries collect chemotherapy data through both passive and active means where possible. Cancer is a reportable disease in all states and the foundation of the surveillance system in the United States was built upon hospital reporting. Hospitals directly collect data on patients receiving chemotherapy at their own facility and additionally attempt to capture chemotherapy on patients receiving some diagnostic and treatment services at their facility who go elsewhere for receipt of medical oncology care. These data are reported electronically to SEER Registries on an ongoing basis (passive collection by SEER). Delivery of chemotherapy outside the hospital setting is often not actively reported and in those situations an attempt is made by SEER staff to collect these data either through remote access to free-standing medical oncology practices, through direct abstracting at the practice, or through other means of follow-back to those facilities (active collection by SEER). This is a resource intensive process, however, that offers enormous challenges as the number of these practices has increased over time. Some registries do receive administrative claims or other electronic files from medical oncology practices as their means of meeting state reporting requirements and a greater emphasis on collecting these data through automated means is in place.

Both of the registries included in our study represent a large proportion of American College of Surgeons Commission on Cancer (CoC) facilities. CoC facilities have invested sizable effort into trying to capture chemotherapy administered outside of the hospital to inform quality measures around standards of care, which may in part explain the low under-ascertainment. [33, 34] Additionally, Georgia was one of the initial pilot states for the Rapid Quality Reporting System (RQRS), a web-based data collection and reporting system that operates in real time and is enabled through the National Cancer Database. Pilot participation in RQRS began in 2008, and the system has been available to all CoC-accredited cancer programs since September 2011. [35] RQRS allows expedited data entry of a critical subset of items specifically relevant to anticipated standard of care treatments, including the receipt of adjuvant chemotherapy for Stage III colorectal cancer. Additionally, RQRS provides alerts prompting participating hospitals to review treatment plans and assure that processes are in place to foster this care, and to help identify demographic variables that may have an impact on the successful delivery of this care. In these ways RQRS has improved both timely capture and more complete reporting of adjuvant chemotherapy for select cancers to population-based registries. [36] Furthermore, studies of the impact of RQRS have found a significant and sustained increase in reported receipt of adjuvant chemotherapy for stage III colorectal cancer among participating sites. [36–38] Whether this increase in documentation of chemotherapy receipt also reflects a change in clinical treatment patterns with actual increased receipt of adjuvant chemotherapy is unknown. It is plausible that the feedback mechanism of RQRS with alerts to participating hospitals and providers may help to prevent patients from “falling through the cracks” by functioning as a cue to provide adjuvant chemotherapy, especially among minority and underserved populations. [37]

In our study, focused on a specific cancer for which well-established quality of care metrics exist, we have shown low rates of under-ascertainment of adjuvant chemotherapy by SEER registries. Perhaps, rather than an “all or none” approach to making chemotherapy data available to researchers, a more nuanced approach could be considered where specific cancers are individually evaluated with respect to treatment ascertainment and cancer-specific datasets are made available. Methods like these would allow for studies of chemotherapy receipt among a more complete population of patients, including those under age 65 and those with non-Medicare insurance.

Another benefit to use of SEER registry data is that receipt of oral chemotherapy agents is recorded. In stage III colorectal cancer oral chemotherapy, namely capecitabine, has become a common component of adjuvant therapy. [39, 40] While capecitabine is covered under Medicare Part B because the same drug is available in injectable form (5-fluoropyrimidine), the ascertainment of capecitabine receipt using data from the durable medical equipment (DME) claims files available with Part B data has been shown to be poor. [41] Furthermore, the majority of oral chemotherapeutic drugs are covered under Medicare Part D. Historically many researchers have not included Part D data in their SEER-Medicare studies of chemotherapy receipt. Because of this, the SEER capture of chemotherapy receipt is perhaps becoming more important as the use of non-intravenous chemotherapy agents grows and use of SEER data could provide a more complete picture of receipt of all chemotherapy agents.

Our study is subject to several limitations inherent to survey research. Analyses were limited by the sample of respondents. We note, however, that the population-based sampling achieved broad demographic representation and the 68% response rate is higher than any previous published cohort of patients with colorectal cancer. [42] The survey relied on respondent report and was thus subject to recall bias, but our reliance on patient reporting permitted individual insights that could not otherwise be obtained. We mitigated recall bias by accepting returned surveys only up until one year after diagnosis. Non-response bias was possible, and those who responded to the survey may have been more likely to receive adjuvant chemotherapy than those who did not respond. It should be noted the results of this study cannot be directly extrapolated to chemotherapy for other cancers, which may have different provider distributions and different treatment patterns from colorectal cancer. Finally, although our sample includes patients from two large and diverse SEER registries that encompass rural to urban areas as well as Southern and Midwestern parts of the United States, our data may not be representative of all SEER registries across the United States. Further studies of chemotherapy ascertainment in additional cancers across different SEER registries are therefore needed.

## Conclusions

While SEER data regarding chemotherapy are available to researchers upon request, it comes with a warning that it is not appropriate for studies of treatment patterns, disparities in receipt of chemotherapy, or comparisons of patient outcomes by receipt of chemotherapy. In a robust study of a diverse population of patients with Stage III colorectal cancer using self-reported receipt of chemotherapy, we have shown a low rate of chemotherapy under-ascertainment among two large SEER registries. In addition, no variation in under-ascertainment was identified by subgroups. Further exploration is needed to determine if the patterns observed in this study carry over to other cancers with defined quality metrics around use of chemotherapy. Use of population-based registry data for select cancers with high chemotherapy ascertainment could be an important resource to researchers investigating modern day chemotherapy receipt and outcomes, providing critically important insight to clinicians and policy makers and informing patient-centered quality improvement initiatives.

## Abbreviations

ACS: American College of Surgeons
CoC: Commission on Cancer
DME: durable medical equipment
ICD: International Classification of Diseases
NCCN: National Comprehensive Cancer Network
RQRS: Rapid Quality Reporting System
SEER: Surveillance, Epidemiology, and End Results
SES: socioeconomic status
